# Migration and Differentiation of Neural Progenitor Cells after Recurrent Laryngeal Nerve Avulsion in Rats

**DOI:** 10.1371/journal.pone.0107288

**Published:** 2014-09-09

**Authors:** Wan Zhao, Wen Xu

**Affiliations:** 1 Department of Otorhinolaryngology-Head Neck Surgery, Beijing Tongren Hospital, Capital Medical University, Beijing, The People's Republic of China; 2 Department of Otorhinolaryngology-Head Neck Surgery, Anhui Provincial Hospital, Anhui Medical University, Hefei, Anhui, The People's Republic of China; Instituto Butantan, Brazil

## Abstract

To investigate migration and differentiation of neural progenitor cells (NPCs) from the ependymal layer to the nucleus ambiguus (NA) after recurrent laryngeal nerve (RLN) avulsion. All of the animals received a CM-DiI injection in the left lateral ventricle. Forty-five adult rats were subjected to a left RLN avulsion injury, and nine rats were used as controls. 5-Bromo-2-deoxyuridine (BrdU) was injected intraperitoneally. Immunohistochemical analyses were performed in the brain stems at different time points after RLN injury. After RLN avulsion, the CM-DiI+ NPCs from the ependymal layer migrated to the lesioned NA. CM-DiI+/GFAP+ astrocytes, CM-DiI+/DCX+ neuroblasts and CM-DiI+/NeuN+ neurons were observed in the migratory stream. However, the ipsilateral NA included only CM-DiI+ astrocytes, not newborn neurons. After RLN avulsion, the NPCs in the ependymal layer of the 4th ventricle or central canal attempt to restore the damaged NA. We first confirm that the migratory stream includes both neurons and glia differentiated from the NPCs. However, only differentiated astrocytes are successfully incorporated into the NA. The presence of both cell types in the migratory process may play a role in repairing RLN injuries.

## Introduction

Recurrent laryngeal nerve (RLN) injury is one of the severe complications induced by thyroid surgery. The incidences of permanent and temporary RLN paralysis were 0.9% and 5.1%, respectively, after thyroidectomy [1]. Patients with RLN injury have a reduced quality of life and experience a variable degree of dysphonia, aspiration and dysphagia [2]. However, it is difficult to treat RLN paralysis. One of the most important factors is the loss of motor neurons in the nucleus ambiguous (NA) caused by RLN injury [3].

Neural stem/progenitor cells (NPCs) are hosted in neurogenic regions. They are self-renewing and can differentiate into neurons, astrocytes and oligodendrocytes [4]. The neurogenic regions in the adult central nervous system (CNS) were first discovered in the subventricular zone (SVZ) of the lateral ventricle and hippocampal dentate gyrus (DG) [5]. The fourth ventricle and central canal in the brainstem and spinal cord [6], [7] were also subsequently identified as neurogenic regions. It has been shown that neurogenesis can be caused by neural injuries and pathological stimuli, including seizures [8], stroke [9] and multiple sclerosis [10]. NPCs also can be induced to migrate long distances to lesioned areas. Therefore, NPCs could be used as reservoirs to regenerate motor neurons after RLN injury.

A previous study suggests that NPCs in the ependymal layer can be activated and induced to migrate to the site of damage following peripheral nerve injuries [11]. Recently, we have further shown that the NPCs in the ependymal layers around the fourth ventricle and central canal in the brainstem contribute to the regeneration of the damaged NA following RLN avulsion [12]. However, the detailed progress of the migration and differentiation of the NPCs from the ependymal layer to the ipsilateral NA after RLN avulsion injury has not yet been addressed. The purpose of this study is to investigate the progress of the NPCs migrating from the ependymal layer to the damaged NA after RLN injury. Furthermore, we address the differentiation and specification of NPCs in the migratory stream and the lesioned NA following RLN injury.

## Materials and Methods

### Animals and surgical procedures

Fifty-four male Sprague-Dawley rats weighing 200–230 g were used in the present study. After the animals were deeply anesthetized by intraperitoneal injection with ketamine (50 mg/kg) and xylazine (10 mg/kg), 20 µl of 0.2% CM-DiI (Molecular Probes, Eugene, OR, USA) in DMSO was injected into the left lateral ventricle (0.8 mm posterior, 1.4 mm lateral of the bregma and 3.7 mm below the dura mater) [13] by stereotaxic injection to label the cells of the ependymal layer.

The animals were randomly divided into two groups: the RLN injury group (n = 45) and the control group (n = 9). Animals in the injury group were subjected to an avulsion injury of the left RLN four days after CM-DiI administration. A vertical skin incision was performed at the neck, and the left RLN was carefully exposed under an operating microscope. At the level of the seventh tracheal ring, the proximal RLN was avulsed and removed from the distal RLN by gentle traction using microhemostat forceps. In the control animals, skin incisions and soft tissue dissections were performed without injury to the nerves.

Beginning on the day of the injury, all of the animals received intraperitoneal injections of 50 mg/kg 5-Bromo-2-deoxyuridine (BrdU, Sigma, St. Louis, MO, USA) dissolved in normal saline twice daily for a maximum of 10 days.

The animals were treated in strict accordance with the National Institute of Health Guide for the Care and Use of Laboratory Animals, and their experimental use was approved by the Animal Ethics Committee of Capital Medical University (personnel No. 15479). All of the surgeries were performed under anesthesia, and all efforts were made to minimize suffering.

### Histological analysis

Injured and control animals were reanesthetized and perfused transcardially with 37°C saline, followed by 4°C 4% paraformaldehyde in PBS at 6 h, 12 h, 1 days, 3 days, 5 days, 7 days, 14 days, 21 days and 28 days after the left RLN injury (n = 5 at each time point). The brainstems were immediately harvested, postfixed for 2 h in 4°C 4% paraformaldehyde in PBS, rinsed in PBS, and kept at 4°C in 30% sucrose in PBS for cryoprotection.

The nucleus ambiguus from each rat was embedded in OCT compound (Sakura, Tokyo, Japan) and serially cryosectioned (14 µm) at −20°C. For immunohistochemistry, the sections were washed 3 times for 15 min with PBS after serial mounting on separate slides and then blocked with PBS containing 10% normal goat serum and 0.3% Triton X-100 (Sigma, St. Louis, MO, USA) for 60 min. Sections were then incubated at 4°C overnight in PBS containing 0.3% Triton X-100 and primary antibodies, as follows: glial fibrillary acidic protein (GFAP, rabbit, polyclonal, 1∶1000, Novus, Littleton, CO, USA), doublecortin (DCX, rabbit, polyclonal, 1∶250, Cell Signaling, Beverly, MA, USA), neuronal nuclei (NeuN, rabbit, polyclonal, 1∶100, Novus, Littleton, CO, USA) and Olig2 (rabbit, polyclonal, 1∶200, Abcam, Cambridge, UK).

For the staining of the proliferating cell marker BrdU, sections were pretreated by incubation with 2 M HCL for 15 min at 37°C, and then the acid was neutralized by immersing the sections in 0.1 M Na_2_B_4_O_7_ for 10 min at room temperature. After 3 washes in PBS, the sections were incubated in 0.05% trypsin for 10 min at 37°C, followed by a rinse with PBS, and then incubation with antisera for BrdU (mouse, monoclonal, 1∶500, Sigma, St. Louis, MO, USA) at 4°C overnight.

Subsequently, all of the sections were washed 3 times in PBS and then incubated with species-specific secondary antibodies diluted in PBS containing 0.3% Triton X-100 at room temperature for 2 h. The following secondary antibodies were used: Alexa 488 goat anti-mouse (1∶500, Molecular Probes, Eugene, OR, USA), Alexa 488 goat anti-rabbit (1∶500, Molecular Probes, Eugene, OR, USA) and Alexa 647 goat anti-rabbit (1∶500, Molecular Probes, Eugene, OR, USA). After 3 washes, the slides were then counterstained with the nuclear marker DAPI (1∶500, Molecular Probes, Eugene, OR, USA). Then, the sections were washed and mounted for microscopy. The Leica TCS SPV laser scanning confocal microscope (Leica Microsystems GMBH, Mannheim, Germany) was used for all analyses.

### Statistical analysis

All data (means ± SD) were analyzed using one-way analysis of variance (ANOVA) for statistical significance, followed by Tukey's post hoc test (GraphPad Software, San Diego, CA, USA). Values of P<.05 were considered statistically significant.

## Results

### Proliferation and migration of NPCs from the ependymal layer to the NA

**Figure 1:**
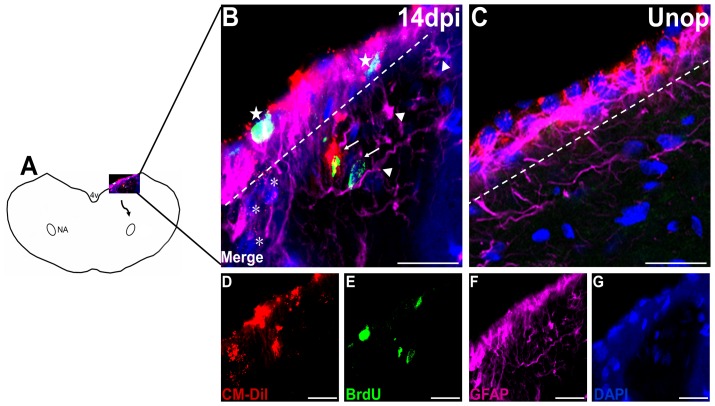
Migration of the CM-DiI+ cells from the ependymal layer after injury. (**A**) Schematic illustration showing the CM-DiI+ cells migrating from the ependymal layer to the ipsilateral nucleus ambiguus (NA) at 14 days after the recurrent laryngeal nerve (RLN) avulsion injury. Animals subjected to RLN avulsion injury (**B**) show BrdU immunoreactivity (green, star) was expressed in the ependymal cells labeled with CM-DiI (red), indicating that the ependymal cells were proliferating, in contrast to the control animals (**C**). CM-DiI+/GFAP+ cells were observed migrating from the ependymal layer, indicating that the neural progenitor cells (NPCs) were migrating from the ependymal layer and differentiating into astrocytes. Some of these cells (arrow) were positive for BrdU (green), indicating that they were proliferating. However, others (arrowhead) showed no immunoreactivity for BrdU. CM-DiI+ cells (asterisk) with neuronal morphology were also observed outside the ipsilateral ependymal layer, indicating that NPCs may be differentiating into neuronal cells while migrating from the ependymal layer. Merged images of (**D**) CM-DiI (red), (**E**) BrdU (green), (**F**) GFAP (violet) and (**G**) DAPI (blue) are shown as (**B**). Bars in A-F =  25 µm. Unop =  unoperated animals; dpi =  days post injury, 4V =  the fourth ventricle, NA =  nucleus ambiguus.

**Figure 2:**
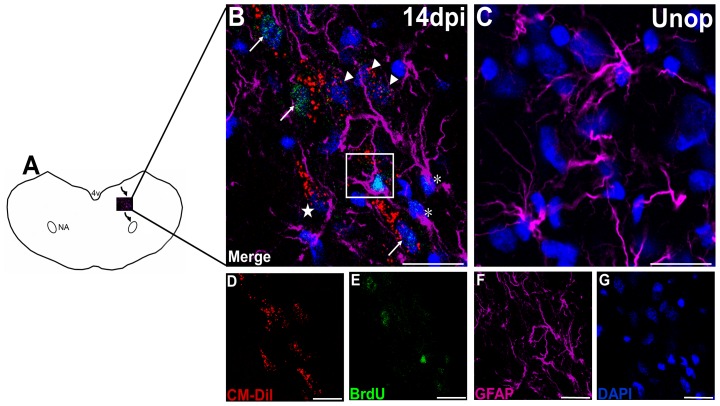
GFAP and BrdU immunoreactivity were seen in the migratory stream. (**A**) Schematic illustration showing the migration routes of the cells from the ependymal layers to the ipsilateral nucleus ambiguus (NA) at 14 days after the recurrent laryngeal nerve (RLN) avulsion injury. (**B**) The CM-DiI+ cells (red) were observed in the region between the ependymal layer and the ipsilateral NA, whereas no migration was observed in the control animals (**C**). A CM-DiI+/GFAP+/BrdU+ cell (white frame) was observed in the migratory stream, indicating that this neural progenitor cell (NPC) was differentiating into an astrocyte and proliferating while migrating to the NA. CM-DiI+/GFAP+ cell (star) can also be observed, indicating that it is not proliferating. Moreover, CM-DiI+ cells with neuronal morphology were observed in the migratory stream, indicating that the NPCs can differentiate into a neuronal fate. Some of the CM-DiI+ cells (arrow) showed BrdU immunoreactivity, indicating that they are proliferating, but others (arrowhead) are not. (**B**) shows the merged image of (**D**) CM-DiI (red), (**E**) BrdU (green), (**F**) GFAP (violet) and (**G**) DAPI (blue). Bars in A-F  = 25 µm. Unop =  unoperated animals; dpi =  days post injury, 4V =  the fourth ventricle, NA =  nucleus ambiguus.

**Figure 3:**
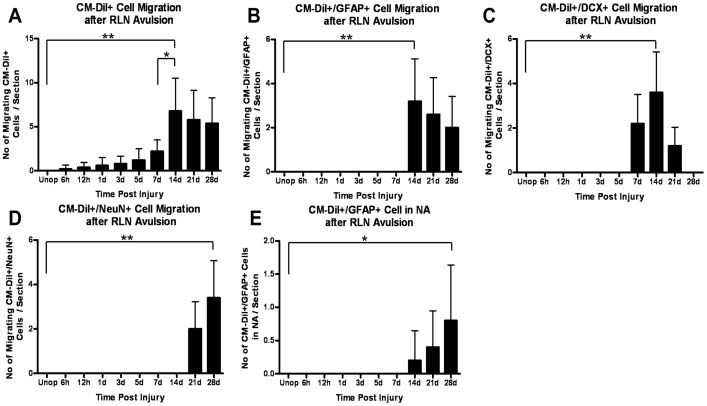
Histogram illustrating the numbers of migrating cells in the ipsilateral brainstem after injury. (**A**) CM-DiI+ cells in the migratory stream increased beginning at 6 h post-injury. The maximal immunoreactivity of CM-DiI was observed at 14 days post-injury, followed by a decline at 28 days post-injury, compared with control animals. (**B**) In contrast to the control animals, the CM-DiI+/GFAP+ cells in the migratory stream were observed at 14 days post-injury. These cells decreased in number in injured animals at later survival times. (**C**) An increase in immunoreactivity of CM-DiI+/DCX+ cells was observed in the migratory stream from 7 to 14 days post-injury and declined from 14 to 28 days after the injury. (**D**) The CM-DiI+/NeuN+ cells in the migratory stream were observed at 21 days post-injury and increased at 28 days post-injury, compared with unoperated animals. (**E**) Quantitation of the CM-DiI+/GFAP+ cells in the NA ipsilateral to the injury showed an increase from 14 to 28 days post-injury. Values are the mean±SD, * *P*<.05, ** *P*<.01. Unop =  unoperated animals.

To evaluate migration, the cells of the ependymal layer were labeled with CM-DiI. The proliferating cells were analyzed with BrdU colabeling. CM-DiI+/BrdU+ cells were observed in the ependymal layer after RLN avulsion injury and in the control animals (Fig. 1), indicating that the NPCs in the ependymal layer were activated and proliferating after the ipsilateral RLN avulsion injury. The CM-DiI+ cells were also observed in the region between the ependymal layer and the ipsilateral NA from 6 h to 28 days post-injury at higher levels than in the control animals (Fig. 1 and Fig. 2). Some cells showed astroglial morphology, and others showed neuronal morphology. BrdU immunoreactivity was detectable in both types of migrating cells. CM-DiI+ cells in the migratory stream increased beginning at 6 h post-injury. The maximal immunoreactivity of CM-DiI in injured animals compared with controls was observed at 14 days post-injury, followed by a decline at 28 days post-injury (Fig. 3 A).

### Differentiation and characterization of the fate of NPCs in the migratory stream to the NA

To characterize the CM-DiI+ cells, we used markers specific for astrocytes, neuronal cells and oligodendrocytes. GFAP is expressed in astrocytes. DCX is expressed in neuroblasts and is also a marker for migrating cells. NeuN is specific to mature neurons. Olig2 is a marker for oligodendrocytes. In this study, we observed that GFAP, DCX and NeuN were co-localized with CM-DiI in the migratory stream following the RLN injury, but Olig2 was not observed to colocalize with CM-DiI.

#### Migrating NPCs differentiate into astrocytes

CM-DiI+/GFAP+ cells were observed outside of the ependymal layer (Fig. 1) and moving away from it (Fig. 2) beginning at 14 days after the RLN avulsion injury. The findings indicated that the NPCs differentiated into astrocytes while migrating from the ependymal layer to the ipsilateral NA. BrdU immunoreactivity was also observed in some of the CM-DiI+/GFAP+ cells, indicating that the migrating astrocytes were proliferating. In the injured animals, CM-DiI+/GFAP+ cells in the migratory stream were observed at 14 days post-injury, and their numbers decreased at later survival times (Fig. 3 B). However, this pattern was not observed in control animals.

GFAP+/BrdU+ cells were also observed in the migratory stream (Fig. 2). The GFAP+ astrocytes were not colocalized with CM-DiI, indicating that they were not differentiated from the NPCs in the ependymal layer. Moreover, BrdU immunoreactivity suggested that they were proliferating. These findings indicate that the dividing astrocytes are reactive inflammatory cells; moreover, the glial reactions around the migratory stream may be helpful in the migration to the NA.

#### Migrating NPCs differentiate into neurons

**Figure 4:**
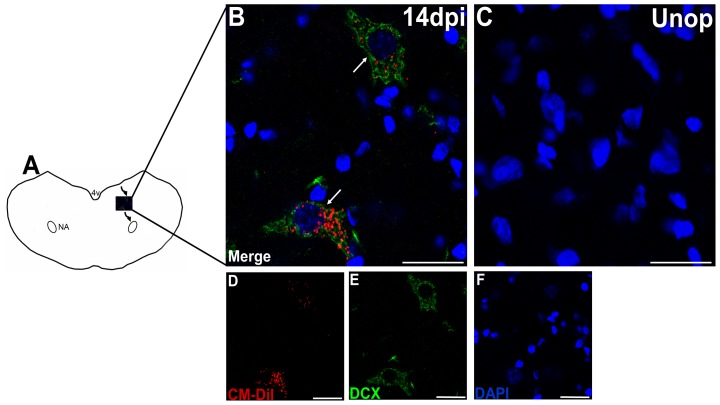
DCX-positive cells were observed in the migratory stream. (**A**) Schematic illustration showing the routes of the cells migrating from the ependymal layers to the ipsilateral nucleus ambiguus (NA) at 14 days after the recurrent laryngeal nerve (RLN) avulsion injury. (**B**) The CM-DiI+/DCX+ cells (arrow) were observed in the migrating stream, but no staining was observed in the control animals (**C**). This indicated that the CM-DiI+ cells with neuronal morphology were differentiating into a neuronal fate. Merged images of (**D**) CM-DiI (red), (**E**) DCX (green) and (**F**) DAPI (blue) are shown as (**B**). Bars in A-E = 25 µm. Unop =  unoperated animals; dpi =  days post injury, 4V =  the fourth ventricle, NA =  nucleus ambiguus.

CM-DiI+ cells with neuronal morphology were observed outside the ependymal layer (Fig. 1) and far along the migrating stream (Fig. 2) after the RLN avulsion injury. Then, we used the specific markers to characterize the CM-DiI+ cells and found that they were immunoreactive with DCX (Fig. 4). DCX is a marker for neuroblasts and migrating cells. The findings suggest that the CM-DiI+ cells with neuronal morphology are migrating NPCs differentiating into a neuronal fate. BrdU immunoreactivity can also be observed in some of the neuroblasts (Fig. 2). CM-DiI+/DCX+ cells were observed at 7 days post-injury. The maximal immunoreactivity of the neuroblasts (3.60±1.82 cells/section) was observed at 14 days post-injury, followed by a decline at 21 days post-injury, and they disappeared by 28 days post-injury when compared with control animals (Fig. 3 C).

**Figure 5:**
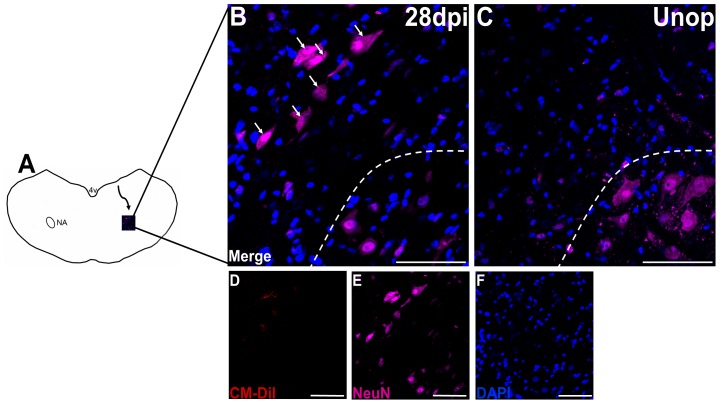
CM-DiI+/NeuN+ cells were seen outside the ipsilateral nucleus ambiguus (NA) at 28 days after injury. (**A**) Schematic illustration showing the routes of the cells migrating from the ependymal layers to the ipsilateral NA at 28 days after the recurrent laryngeal nerve (RLN) avulsion injury. (**B**) A decrease in the number of motor neurons stained with NeuN was observed in animals with RLN avulsion injuries at 28 days post-injury (dotted circle), in contrast to the control animals(**C**). CM-DiI+/NeuN+ cells (arrow) were observed outside the ipsilateral NA at 28 days post-injury (**B**), compared with the control animals (**C**). This indicated that the neural progenitor cells (NPCs) were differentiating into neurons, but they did not reach the ipsilateral NA. Merged images of (**D**) CM-DiI (red), (**E**) NeuN(violet) and (**F**) DAPI (blue) are shown as (**B**). Bars in A-E = 75 µm. Unop =  unoperated animals; dpi =  days post injury, 4V =  the fourth ventricle, NA =  nucleus ambiguus.

In contrast to the control animals, a decrease in the number of motor neurons stained with NeuN was observed in animals with RLN avulsion injuries at 28 days post-injury (Fig. 5). CM-DiI+/NeuN+ cells were also observed far away from the ipsilateral NA at 21 days (2.00±1.22) post-injury, followed by an increase in cells far from the ipsilateral NA at 28 days (3.40±1.67) post-injury (Fig. 5 and Fig. 3 D). The findings suggest that the migrating neuroblasts eventually differentiated into mature neurons, but the new-born neurons did not reach the ipsilateral NA to replace the cells lost from the NA.

### NPCs differentiate into astrocytes in the ipsilateral NA

**Figure 6:**
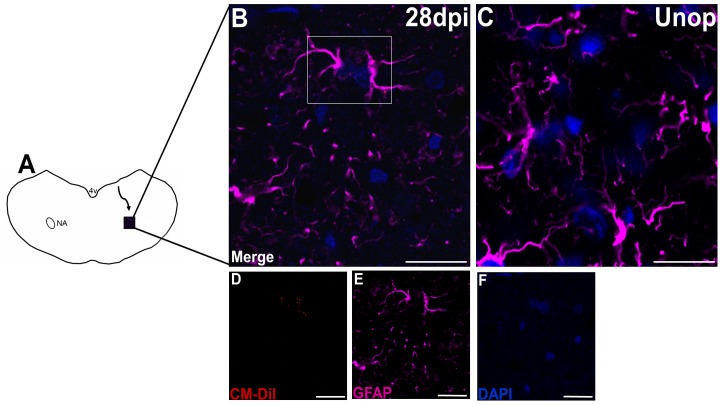
CM-DiI+/GFAP+ cells were observed in the ipsilateral nucleus ambiguus (NA) at 28 days after injury. (**A**) Schematic illustration showing the routes of the cells migrating from the ependymal layers to the ipsilateral NA at 28 days after the recurrent laryngeal nerve (RLN) avulsion injury. (**B**) The CM-DiI+/GFAP+ cells (white frame) were observed in the ipsilateral NA at 28 days post-injury, which was not observed in the control animals (**C**). The (**D**) CM-DiI-labeled (red) cells were colabeled with (**E**) GFAP (violet) and (**F**) DAPI(blue), indicating that the neural progenitor cells migrated into the ipsilateral NA and differentiated into astrocytes. Bars in A-E = 25 µm. Unop =  unoperated animals; dpi =  days post injury, 4V =  the fourth ventricle, NA =  nucleus ambiguus.

The migrating CM-DiI+ cells in the NA showed colabeling with GFAP but were negative for nestin, DCX, NeuN and Olig2 (Fig. 6). These findings indicate that the migrating NPCs were differentiating into astrocytes, but not neurons or oligodendrocytes, in the ipsilateral NA. The migrating astrocytes appeared in the ipsilateral NA at 14 days post-injury. Although there was an increase in CM-DiI+/GFAP+ cells in the NA ipsilateral to the injury from 14 to 28 days post-injury (Fig. 3E), the numbers of CM-DiI+ astrocytes in the ipsilateral NA after injury remained low.

## Discussion

Neurogenesis in the adult mammalian brain has been demonstrated to occur in response to neural injuries and neurologic degenerative disorders. Endogenous NPCs can be induced to proliferate, migrate, differentiate, survive and compensate for cell loss in the area of a neural lesion. However, the underlying mechanism remains unclear. Previous reports suggest that NPCs in the ependymal layer can be activated and migrate to the site of damage after various neural diseases, including spinal cord injury [7], the EAE-model of multiple sclerosis [10] and hypoglossal nerve avulsion [11]. Nevertheless, the detailed progress of the migration and differentiation of the NPCs from the ependymal layer to the lesioned NA after RLN avulsion injury has not previously been addressed.

This study shows that the NPCs in the ependymal layers of injured but not control animals were activated and that they proliferated and migrated to the ipsilateral NA from 6 h to 28 days post-injury. These findings suggest that the endogenous NPCs attempt to repair the lesioned NA. It is generally known that a retrograde axonal reaction can be caused by injury of a peripheral motor nerve [14]. Decreased motor neuron survival numbers, reactive microglia, astrocytes and macrophages were observed after the RLN avulsion injury in our previous report [12]. The ability of activated astrocytes and microglia to direct the NPCs to migrate to the destination has been previously proved [15]. Microglia can release chemokines to attract the migrating NPCs after brain injury, and astrocytes can upregulate these potent attractants for migrating NPCs [16]. In addition, studies have also demonstrated neuroblast chain migration through astrocytic tubes after brain injury [8]. Similarly, some proliferating glial cells can be observed around the migratory stream in our study. Therefore, we propose that the recruitment of the ependymal layer-derived NPCs may be due to the activation of microglia and astrocytes.

Interestingly, our data showed that the maximal immunoreactivity of CM-DiI was observed at 14 days post-injury. This result is consistent with the appearance of the CM-DiI+/GFAP+ cells and the highest numbers of CM-DiI+/DCX+ cells, also at 14 days post-injury. Furthermore, the quantitation of CM-DiI+ cells in the migratory stream showed a decrease from 14 to 28 days post-injury. This pattern can be explained as follows. First, the inflammatory response in the ipsilateral NA decreases; moreover, the ability of the inflammatory cells to release the attractive chemokines decreases by later time points. Second, the NPCs are speculated to release anti-inflammatory factors to halt their own migration to the destination.

This study first observed the differentiation in neuronal fate of NPCs in the migratory stream following the RLN avulsion injury. The CM-DiI+/DCX+ cells were observed in the migratory stream from 7 to 28 days post-injury; moreover, the CM-DiI+/NeuN+ cells appeared in the migratory stream at 21 days post-injury and increased at 28 days post-injury, compared with unoperated animals. DCX has been shown to be essential for microtubule stabilization in neuroblasts [17] as well as proper neurogenesis [18] and neuronal migration [19]. DCX is specific to neural precursor cells and migrating cells, whereas NeuN is expressed in mature neurons. Therefore, the appearance of CM-DiI+/DCX+ cells and CM-DiI+/NeuN+ cells in the migration stream provides strong evidence that the migrating NPCs differentiated into neurons. Previous studies have reported the neuronal differentiation of NPCs following various CNS lesions [7], [11]. In addition, both neuronal lineage differentiation and glial differentiation in NPCs can be induced by the endogenous factors released by the inflammatory response and the extracellular matrix, such as cytokines, chemokines, neurotrophic factors, growth factors and morphogens [20].

We are particularly interested to note that the newborn neurons failed to compensate for the cells lost from the damaged NA. The CM-DiI-labeled NeuN+ cells were observed far from the lesioned NA at the final timepoint. We speculate that this may have occurred for the following reasons. First, the astrocytes around the lesioned NA may have formed a “glial scar” as part of the inflammatory response, inhibiting the migrating neurons from moving closer. Second, the niche in the ipsilateral NA may have changed in accordance with the RLN injury, making it inhospitable for the newborn neurons. Third, we focused on the response of ependymal NPCs during a limited time period post-injury, which may be too short to observe the arrival of the migrating newborn neurons in the damaged NA.

Apart from observing the neuronal lineage differentiation, we observed the CM-DiI+/GFAP+ cells in the migration stream from 14 to 28 days post-injury. This observation coincides with that from a study by Mothe and Tator [21], who found DiI-labeled GFAP+ ependymal cells at 14 days after minimal injury. Similarly, another study [7] showed that the migrating ependymal cells differentiated into astrocytes at 28 days following a dorsal funiculus incision.

It is interesting to observe that only CM-DiI+ astrocytes but not CM-DiI+ neurons appeared in the damaged NA. It suggests that the niche in the lesioned NA may be appropriate for astroglial differentiation. In addition, a previous study reported that DCX+ precursors could be redirected from neuronal to glial fate under some pathological conditions [22]. Moreover, Gordon et al [23] demonstrated that the SVZ temporarily generated DCX+ neuroblasts, followed by migrating cells showing a glial phenotype in the striatum following quinolinic acid lesioning. We speculate that the CM-DiI+/GFAP+ astrocytes in the lesion NA may be derived from the CM-DiI+/DCX+ precursor cells in the migratory stream. Indeed, very few CM-DiI+ astrocytes were observed in the NA after the RLN injury, indicating that both neuronal and glial differentiation may be suppressed in the NA after injury.

## Conclusion

In this study, we first confirmed that NPCs in the migratory stream to the lesion NA differentiate into both neurons and glia. However, the newborn neurons did not reach the damaged NA to integrate into the neural circuit. Instead, the NPCs in the ipsilateral NA differentiated only into astrocytes. An increase in the number of the neuronal lineage cells in the lesioned NA may be an important goal for successful therapies for RLN injury. The detailed molecular mechanism directing the endogenous neurogenesis after RLN injury should be further studied. These findings will contribute to functional neural recovery and to future possibilities for the treatment of laryngeal paralysis.
